# Protein Supplementation Improves Performance of Lambs Fed Low-Quality Forage

**DOI:** 10.3390/ani10010051

**Published:** 2019-12-25

**Authors:** Belal S. Obeidat, Hadil S. Subih, Mysaa Ata

**Affiliations:** 1 Department of Animal Production, Faculty of Agriculture, Jordan University of Science and Technology, Irbid 22110, Jordan; 2 Department of Nutrition and Food Technology, Faculty of Agriculture, Jordan University of Science and Technology, Irbid 22110, Jordan; hssubih@just.edu.jo; 3 Department of Animal Production and Protection, Faculty of Agriculture, Jerash University, Jerash 26150, Jordan; mysaata@jpu.edu.jo

**Keywords:** growth performance, digestibility, intake, soybean meal

## Abstract

**Simple Summary:**

In Jordan and in the Middle East, both quantity and quality of forage have declined in recent decades. Livestock producers have thus tended to use plant protein supplements such as soybean meal to correct for protein deficiencies. In this study, soybean meal supplementation improved the performance of lambs fed low-quality forage.

**Abstract:**

The objective was to investigate the effect of supplementing Awassi lambs fed low-quality forage with soybean meal. Twenty-one lambs (initial body weight (BW) of 26.1 ± 2.57 kg) were randomly assigned to the study diets, 1) the basal diet (forage mix; CON; n = 7); 2) the basal diet supplemented with either 125 (SBM125; n = 7); or 3) with 250 (SBM250; n = 7) SBM g/head/day. The forage mix was composed of 65% wheat straw and 35% alfalfa hay. The experimental diet was offered to the animals for 54 days. On day 40, four animals from each group were chosen to assess N balance and nutrient digestibility. The intake of crude protein (CP) and dry matter (DM) was the highest (*p* < 0.016) in the SBM250 group, followed by the SBM125 group and the CON diet. Digestibility of DM and CP was higher (*p* <0.05) for the SBM-containing diets than the CON diet. Final BW and average daily gain were greater (*p* < 0.008) for lambs that consumed SBM-containing diets than for those that consumed the CON diet. In summary, the growth performance, forage utilization, and nutrient digestibility improved for lambs fed low-quality forage when supplemented with soybean meal.

## 1. Introduction

Jordan is classified as a semi-arid country and receives around 200 mm of rainfall per year. Most of the rainfall occurs in December and January, and the pasture quality and quantity peak in February and March (late winter and early spring) [1]. In recent decades, however, erratic rainfall and overgrazing have caused a decline in the quality and quantity of high-quality forages. Low-quality forages are defined as those forages that are deficient in crude protein (less than 8% crude protein; CP), low in soluble sugars and starches, and are comprised mostly of natural pastures or crop residues. The diets of ruminants under grazing are composed mainly of low-quality forages throughout most, if not all, of the year, and this leads to the elimination of grazing areas and the reduction of grazing performance.

Lambs average daily gains finished in forage is usually lower than in lambs finished on high grain diets [2,3]. The use of supplemental protein can enhance the establishment of lambs’ daily nutrient requirements and increase the growth rates during consumption periods of low-quality forage [4]. The consumption of low-quality forage by ruminants has been found to be enhanced by supplementation with ruminally degradable protein [5]. Furthermore, the addition of CP to the low-quality forage offered to ruminant animals can reduce feeding expenses while maintaining or enhancing animal performance and the intake and digestibility of nutrients [6,7]. To improve the productivity of livestock grazing on low-quality forages, protein supplements such as soybean meal (SBM) are used in Jordan and worldwide [8]. Ramos et al. [9] reported that the growth performance was improved in lambs receiving supplemental graded levels of protein (i.e., 12%, 16%, or 20% CP) compared with lambs not fed supplemental protein when grazing low-quality forages. Since the cost of SBM is considered to be high compared to other feedstuffs, livestock producers use SMB in small amounts to enhance and maintain livestock performance and production [10].

The study hypothesis was that supplementation with SBM would improve forage intake and utilization, nutrient digestibility, and N balance as well as growth performance in lambs fed low-quality forages. The aims of this study were to investigate the influences of protein supplementation on performance, nutrient intake and digestibility, and the rumen pH of Awassi lambs fed low-quality forage.

## 2. Materials and Methods

This study was performed at the Agricultural Research and Training Unit (ARTU) at Jordan University of Science and Technology (JUST). The study’s method and procedures were approved by the JUST Institutional Animal Care and Use Committee (Protocol #: 16/03/03/375).

Twenty-one Awassi lambs (26.1 ± 2.57 kg of body weight; 4–5 months of age) were assigned randomly into 3 groups, each of which were fed a different quantity level of SBM. Three levels of soybean meal (SBM; protein supplements) were 0 g SBM per head, per day (CON; n = 7), 125 g SBM per head, per day (SBM125; n = 7), or 250 g SBM per head, per day (SBM 250; n = 7). All of the lambs were fed a diet of low-quality forage (mix of 65% wheat straw and 35% Alfalfa hay). The composition of the forage mix was 953, 77.5, 668, and 439 g/kg of dietary dry matter (DM), crude protein (CP), neutral detergent fiber (NDF) and acid detergent fiber (ADF), respectively.

Lambs were separated and housed in individual pens (1.5 × 0.75 m). The study continued for 54 days, 7 of which were used to adapt the animals to the pens and 47 of which comprised the test feeding period. Feed was provided daily at 8:30 a.m. on an ad libitum basis to evaluate nutrient intake. Refusals were collected, weighed, and recorded daily. Lambs were given unlimited access to fresh water throughout the study. To evaluate the average daily gain (ADG), lambs were weighed at the commencement of the study and subsequently every 2 weeks.

At the end of the experiment, 12 lambs were randomly selected and placed in metabolic cages (0.8 × 1.05 m) to collect animal feces and urine. After an acclimation period of 5 days, the lambs’ feces and urine were collected for 5 more days. The feces were weighed and stored (10%) at −20 °C. At the same time as the feces collection, urine was collected in plastic containers containing 50 mL 1 N HCl. Urine samples were weighed and recorded, and 5% of each was kept for N analysis. Fecal samples were combined for each lamb, dried in an oven at 55 °C, ground, and kept for further chemical analyses. Samples of feces and feeds were analyzed for DM (at 100 °C in the oven for 24 h), CP (Kjeldahl procedure; 6.25 × N) using procedures established by AOAC [11]. Fiber contents (NDF and ADF) were analyzed using Van Soest’s [12] ANKOM 2000 procedure. Urine was analyzed for N using the Kjeldahl procedure.

On days 0, 11, 22, 33, and 44, blood samples were drawn from the jugular vein in plain vacutainers at 800 h (before feeding). Blood samples were centrifuged after 1 h at 3000 rpm for 15 min. Serum samples were immediately separated, transported, and stored in the lab at −20 °C until the day the glucose and urea N analyses were to take place. The serum glucose and urea N content were analyzed using a spectrophotometer and commercial kits in accordance with the manufacturer’s terms and conditions. On the last day of the study and following the offer of feed, 5 mL of rumen fluid were collected to evaluate rumen fluid pH using a syringe fitted with a G14 needle at 0, 2, and 4 h.

### Statistical Analysis

All data were analyzed using a mixed procedure of SAS with the lambs representing the random effect and the diets representing the fixed effect. Serum glucose and urea N content were analyzed as repeated measures using diet, day, and interaction as fixed effects. The significant differences were considered at (*p* < 0.05).

## 3. Results

Forage consumption was greater (*p* < 0.05) in the groups supplemented with SBM than in those on the CON diet alone (Table 1). The intake (g/day) of DM and CP was highest (*p* < 0.016) in the SBM250 diet, followed by the SBM125 diet, and was lowest with the CON diet. Lambs fed the SBM125 and SBM250 diets had greater (*p* ≤ 0.028) intake of NDF and ADF compared to the lambs fed the CON diet.

The digestibility of DM and CP was greater (*p* < 0.05) in SBM-containing diets compared to the CON diet (Table 2). The digestibility of NDF was higher (*p* = 0.029) in the SBM250 diet than in the CON diet, whereas the digestibility of the SBM125 diet was similar to that of the other two diets. Nitrogen excreted in feces and urine (g/day) was shown to be lower (*p* < 0.05) for lambs that had consumed the CON and SBM125 diets compared to lambs that had consumed the SBM250 diet. Retained N (g/day) was found to be the highest (*p* ≤ 0.015) for lambs that consumed SBM250 as compared to lambs that had consumed the CON and SBM250 diets. Nitrogen retention (g/100 g) was greater (*p* ≤ 0.002) in the SBM125 and SBM250 diets compared with the CON diet.

Initial body weight for the different groups of lambs was similar among dietary treatments (Table 3). Final BW and ADG were greater (*p* ≤ 0.008) for lambs that consumed the SBM vs. the CON diet. Over the course of the study, no diet × day effects (*p* ≥ 0.09) were observed for serum concentration of the glucose and urea N. However, serum concentrations of glucose were greater for the SBM diets compared to the CON diet. Serum glucose (*p* = 0.0003) and urea N (*p* = 0.0024) content were greater in the lambs that consumed the SBM125 and SBM250 diets compared to those that consumed the CON diet.

**Figure 1:**
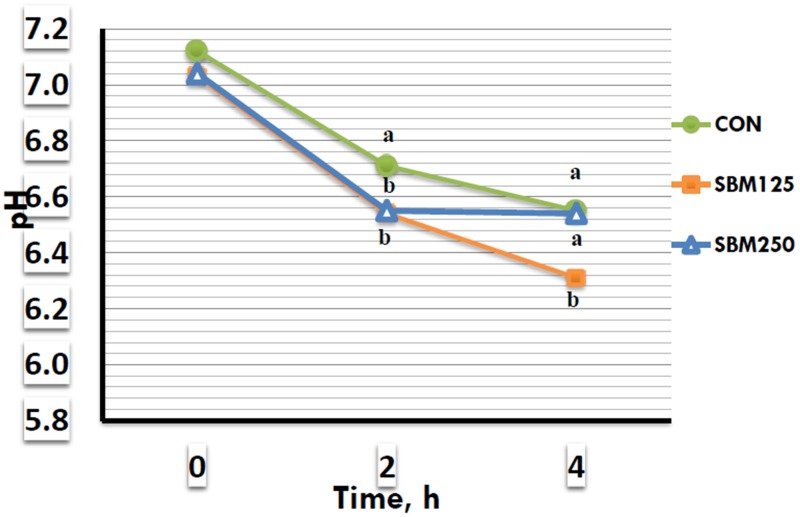
Effect of protein supplementation on rumen fluid pH of Awassi lambs fed low-quality forage diets.

An interaction between the treatment × time (*p* < 0.0001) was detected for rumen fluid pH among the diets (Figure 1). Rumen pH was similar for lambs fed the different diets at 0 h. Lambs that consumed the SBM125 and SBM250 diets had lower rumen pH after 2 h of feeding than the lambs fed the CON diet. After 4 h of feeding, the lambs that consumed the SBM125 diet showed lower pH than the lambs fed both CON and SBM250 diets.

## 4. Discussion

The nutritional pastures in Jordan do not provide sufficient protein content to meet the growth needs of lambs. This study proved the need to support grazing animals with protein. Regardless of the lambs’ performance, the current data support the hypothesis that lambs supplemented with CP will have greater feed intake and growth compared to the non-supplemented lambs grazing low-quality forages. In the present study, the addition of SBM for growing lambs that consumed low-quality forage diets enhanced forage intake by 20–27%, improved digestion by 15%, and improved the N balance of low-quality forage by 40% and growth performance. In the current study, the supplementation of SBM stimulated forage intake comparable to the results of Paulino et al. [13]. McDonald et al. [14] revealed that there is a positive relationship between protein intake and the digestibility of feed. In harmony with previous studies, a positive interaction effect on nutritional quality and utilization was found between low-quality forages and protein supplementation. On the other hand, this positive response to intake was not consistent amongst different sources of protein supplements.

Figueiras et al. [15] reported that increased forage intake can be reached by maintaining a balanced protein-to-energy ratio in the diet, while the supplementation of protein can improve dietary equilibrium. This can reduce the metabolic discomfort of the animal and result in greater forage intake. The intake of DM and CP was greater for lambs supplemented with 250 g of SBM when compared to the other groups, while intake of fiber was greater for lambs that consumed SBM125 and SBM250 than for those in the CON group. Conversely, Bohnert et al. [16] reported that total DM intake increased with protein supplementation. Moura et al. [17] reported no differences in nutrient (i.e., DM, CP, or NDF) intake for lambs that consumed diets with supplemental protein. The researcher inferred this to be due to the high NDF content of hay, which may have diluted the effects of the concentrated feed used. Moreover, Schauer et al. [18] concluded that the consumption of NDF greater than 12.5g/kg BW per day, would not improve DM intake even with the addition of protein as a supplement to the diets.

In this study, the digestibility of nutrients was improved by the addition of SBM. Offering diets with CP supplementation had improved the intake and passage rate of nutrients in response to the increase of the available nitrogen supply in the rumen. In addition, increasing CP intake had a positive effect on the digestibility of fiber. This is due to the enhancement of rumen activities for the fermentation of fiber. In the current study, the increase of hay intake may have increased the passage rate as well as the time needed by rumen microbes to access substrates indicating the positive response on digestibility due to protein supplementation. However, other studies have reported that supplementation of protein has no effect on nutrient digestibility [17,19,20]. They attributed that to the low-quality forage source and small particle size of the ration ingredients. This might also be attributed to the palatability of the diet fed, CP content and intake, high DM intake, better digestibility of the diet, and the best nitrogen utilization.

Nitrogen balance in our study was affected by the addition of SBM. This result agrees with what was reported by other researchers [6,16,21]. They attributed low N digestibility to the greater fiber and lower CP content of the forage. Moreover, they reported that N in feces that is not directly from the diet can represent a portion of the total fecal N in non-supplemented animals, causing low N digestibility and intake with livestock fed low-quality forages. Furthermore, Moura et al. [17] reported that fecal N and urine N as a function of the total N intake increased by 30% for lambs fed supplemented diets compared to the control group. This might be related to when protein degradation exceeds the rate of carbohydrate fermentation, large amounts of nitrogen compounds can be excreted via urine and feces. [22]

The lambs’ performance was improved by the addition of SMB to their diets. These results agree with those reached by other researchers [20,23] who reported that the ADG and the BW gain of different sheep breeds that consumed higher levels of protein supplements were enhanced by protein supplementation. In this study, the increase in the lambs’ performance might be related to a moderately greater nutrient availability combined with higher supplement levels of SBM in their diets. Blood glucose and urea N was increased with the lambs that were fed SBM. Other studies reported results in conflict with those of this study [24,25], as they found that blood urea and glucose were reduced in cattle and lambs that consumed diets supplemented with different types of soybeans. The current study finding might be attributed to the higher availability and digestibility of nutrients, which might have caused elevated serum sugar and urea levels.

A reduction in rumen fluid pH was noticed by extending the time of rumen fermentation to the different diets offered to lambs. The study by Moura et al. [17] provided results similar to those of this study. More alkaline pH in the rumen fluid was recorded before a high amount of nutrients was available to the rumen microbial fermentation. However, the decrease in ruminal pH after the beginning of feeding might be explained by the amount of carbohydrates entering the rumen followed the production of short-chain fatty acids, and by that, the reduction of pH [26]. On the other hand, the pH values did not fluctuate and stayed within the ruminal pH rang after 6 hours of feeding protein supplements, as reported by Woyengo et al. [22].

## 5. Conclusions

Supplementing protein (soybean meal) increased hay intake and average daily gain in lambs fed low-quality forage compared with the lambs not supplemented. The results herein demonstrate that the growth performance, forage utilization, digestibility of nutrients, and nitrogen balance were improved in lambs fed low-quality forages when supplemented with soybean meal than the control diet. In conclusion, it is recommended to supplement animals fed on low-quality forage with soybean meal or other protein sources.

## Figures and Tables

**Table 1:** Effect of protein supplementation on nutrient intake of Awassi lambs fed low-quality forages.

Item	Diets ^1^			SEM	*p*-Value
	CON	SBM125	SBM250		
Nutrient intake, g/day					
Hay intake	577 ^a^	717 ^b^	789 ^b^	49.4	≤0.05
Dry matter	577 ^a^	831 ^b^	1017 ^c^	49.4	≤0.016
Crude protein	45 ^a^	111 ^b^	171 ^c^	3.8	<0.0001
Neutral detergent fiber	385 ^a^	497 ^b^	562 ^b^	33.0	≤0.028
Acid detergent fiber	253 ^a^	327 ^b^	371 ^b^	21.7	≤0.027

^1^ Diets: 0 g soybean meal (SBM) per head per day (CON; n = 7), 125 g SBM per head per day (SBM125; n = 7) or 250 g SBM per head per day (SBM 250; n = 7). ^a,b,c^ Within a row means without a common superscript differ (*p* < 0.05).

**Table 2:** Effect of protein supplementation on nutrient digestibility and N balance of Awassi lambs fed low-quality forages.

Item	Diets ^1^			SEM	*p*-Value
	CON	SBM125	SBM250		
Digestibility coefficients					
Dry matter	53.8 ^a^	64.0 ^b^	62.1 ^b^	2.71	≤0.05
Crude protein	52.6 ^a^	76.2 ^b^	77.4 ^b^	1.19	<0.0001
Neutral detergent fiber	52.4 ^a^	61.3 ^ab^	65.4 ^b^	3.54	=0.029
N Balance					
N intake, g/day	9.0 ^a^	19.4 ^b^	26.9 ^c^	0.92	≤0.0003
N feces, g/day	4.27 ^a^	4.59 ^a^	6.10 ^b^	0.346	≤0.013
N urine, g/day	1.98 ^a^	4.62 ^ab^	7.03 ^b^	1.188	≤0.015
N retained, g/day	2.75 ^a^	10.17 ^b^	13.79 ^c^	0.855	≤0.015
N retention, g/100 g	31.04 ^a^	52.48 ^b^	51.88 ^b^	5.205	≤0.020

^1^ Diets: 0 g SBM per head per day (CON; n = 7), 125 g SBM per head per day (SBM125; n = 7) or 250 g SBM per head per day (SBM 250; n = 7). ^a,b,c^ Within a row, means without a common superscript differ (*p* < 0.05).

**Table 3:** Effect of protein supplementation on growth performance and blood metabolites of Awassi lambs fed low-quality forages.

Item	Diets ^1^			SEM		*p*-Value
	CON	SBM125	SBM250			
Initial body weight (kg)	26.8	25.3	26.3		1.14	≥0.3573
Final body weight (kg)	23.6 ^a^	28.1 ^b^	30.8 ^b^		1.32	<0.0076
Total gain (kg)	−3.2 ^a^	2.8 ^b^	4.5 ^b^		0.068	<0.0001
Average daily gain (g)	−67.4 ^b^	59.3 ^a^	95.8 ^a^		14.56	<0.0001
Glucose, mg/dL	44.6 ^a^	60.4 ^b^	61.8 ^b^		2.65	0.0003
Urea N	19.6 ^a^	27.0 ^b^	26.1 ^b^		1.38	0.002

^1^ Diets: 0 g SBM per head per day (CON; n = 7), 125 g SBM per head per day (SBM125; n = 7) or 250 g SBM per head per day (SBM 250; n = 7). ^a,b^ Within a row means without a common superscript differ (*p* < 0.05).
